# Association between type 2 diabetes (T2D) and tooth loss: a systematic review and meta-analysis

**DOI:** 10.1186/s12902-022-01012-8

**Published:** 2022-04-13

**Authors:** Amir Reza Ahmadinia, Donya Rahebi, Masoud Mohammadi, Mousa Ghelichi-Ghojogh, Alireza Jafari, Firooz Esmaielzadeh, Abdolhalim Rajabi

**Affiliations:** 1 Dental Research Center, School of Dentistry Golestan University of Medical Sciences, Gorgan, Iran; 2 Research Center of Gastroenterology and Hepatology, Golestan University of Medical Sciences, Gorgan, Iran; 3 Department of Health Management and Social Development Research Center, Golestan University of Medical Sciences, Gorgan, Iran; 4 Department of Health Education and Health Promotion, School of Health, Social Development and Health Promotion Research Center, Gonabad University of Medical Sciences, Gonabad, Iran; 5 School of Nursing and Allied Medical Sciences, Maragheh University of Medical Sciences, Maragheh, Iran; 6 Health Management and Social Development Research Center, Faculty of Health, Golestan University of Medical Sciences, Gorgan, Iran

**Keywords:** Type 2 diabetes, Tooth loss, Periodontitis, Meta-analysis, Epidemiologic studies

## Abstract

**Background:**

Several studies have assessed the relationship between type 2 diabetes (T2D) and tooth loss; however, results have been inconsistent. Therefore, the present systematic review and meta-analysis of observational studies was designed to examine the association between T2D and tooth loss.

**Methods:**

This systematic review and meta-analysis was conducted based on Preferred Reporting Items for Systematic Reviews and Meta-Analyses (PRISMA) Guideline. We searched all the relevant studies in international databases of Scopus, PubMed, ProQuest, Web of Science, Cochrane Library, and Google scholar search engine until February 2022. The heterogeneity of the studies was calculated using the I^2^ index. Measure of effect and 95% confidence interval (CI) were extracted from each study. The results of the study were analyzed using the random effects model.

**Results:**

In the present study, 22 eligible studies were included. Meta-analysis of unadjusted and adjusted results showed that T2D significantly increased the risk of tooth loss, and Odds Ratio (OR) unadjusted was 1.87 (95% CI: 1.62–2.13, *p* < 0.001), and OR adjusted was 1.20 (95% CI: 1.10–1.30, *p* < 0.001), respectively. Subgroup analysis based on study design for adjusted OR indicated that in the cohort study (OR: 1.29, 95% CI: 1.07–1.51), in the cross-sectional study (OR: 1.15, 95% CI: 1.06–1.23), and in the case-control study (OR: 5.10, 95% CI: 1.01–9.18) there was a significant association between T2D and tooth loss. Other subgroups analyses showed consistent results and no publication bias existed.

**Conclusions:**

The findings suggest that T2D is associated with increased risk of tooth loss. This conclusion may provide useful evidence for correlated clinical researches.

**Supplementary Information:**

The online version contains supplementary material available at 10.1186/s12902-022-01012-8.

## Introduction

Diabetes mellitus is a chronic metabolic disease characterized by hyperglycemia caused by defects in insulin secretion, insulin function, or both. It is divided into two types: type 1 and type 2 diabetes [1, 2]. Diabetes mellitus is a global health problem affecting 463 million people aged 20–79 years in 2019, projected to rise to 700 million by 2045 [3]. The estimated global direct health expenditure on diabetes in 2019 is United States dollar (USD) 760 billion and is expected to grow to a projected USD 825 billion by 2030 and USD 845 billion by 2045. There is a wide variation in annual health expenditures on diabetes [4]. About one-third of people with diabetes are unaware of their illness, and many go undiagnosed until the onset of symptoms [5]. Diabetes has complications such as retinopathy, neuropathy, microvascular and macrovascular changes, and oral complications such as tooth decay, periodontal disease, dry mouth, and tooth loss [2, 6]. Diabetes increases the risk of oral disease directly (e.g., gingival inflammatory response) and indirectly (e.g., decreased saliva production due to medication) [7]. About one-third of people with diabetes have severe periodontal disease (periodontitis) or severe gum disease In people with diabetes, periodontal disease eventually leads to the loss of one or more teeth [8]. Indeed, periodontitis, also known as gum disease, is a serious infection of the gums that can damage soft tissue and, if left untreated, the bone that supports your teeth. Periodontitis can cause teeth to loosen or cause teeth loss [9]. In fact, the American Dental Association has published a new study that believes that one in five cases of tooth loss is related to diabetes [10]. In addition, most of these studies [11–13] showed that very few patients diagnosed with diabetes visit their dentist regularly for periodontal exams, and many patients are unaware of the impact of diabetes on oral health. They do not even know that diabetes can cause tooth loss [14]. Severe tooth decay and chronic periodontal disease are the main causes of tooth loss in adults [15, 16]. The severity and prevalence of tooth loss is still a major problem in many countries around the world [17]. According to the National Health and Nutrition Examination Survey (NHANES), the prevalence of edentulous among people aged 60 and older was 31 and 25% between 1988 and 1994 and 1999–2002, respectively. In addition, the average number of teeth in the mouth of people aged 60 and over during these years was 18.4 and 19.4, respectively [18]. Although the prevalence of tooth loss has decreased over the past few decades, it is still a major public health problem [7]. Tooth loss and complete edentulous are both poor health outcomes that negatively affect a person’s quality of life [18, 19]. Elderly people with diabetes have more teeth lost and a lower quality of life than non-diabetics [20].

Oral health is a very important factor in increasing the quality of life despite increasing attention and dental care, various risk factors such as: age, socioeconomic status [7, 18, 21], gender, race and ethnicity [19], level of education, family income, geographical location, access to care, history of smoking, insurance [18, 22], lifestyle, oral hygiene habits and regular visits to the dentist [21]. Adverse effects of tooth loss include: difficulty chewing, difficulty speaking, difficulty smiling, cosmetic problems, negative social points such as interacting with others, and social isolation [7, 18]. Absence of fiber and vegetables and eating more cholesterol and fatty foods [6, 23] or high-carbohydrate diets [19], can reduce cognitive function and increase vulnerability to serious and fatal infections [7, 24]. The findings suggest that tooth loss is independently associated with chronic diseases such as cardiovascular disease, hypertension, stroke, cancer and other systemic diseases [19, 25]. The increased incidence of oral health problems may be due to the rapid increase in T2D and obesity in adults, especially in African Americans [7]. Several studies have evaluated the relationship between T2D and tooth loss; however, the results have been inconsistent [26–30]. Although there have been meta-analyses on the relationship between T2D and periodontitis that have evaluated predictors of tooth loss in patients with periodontitis, including Diabetes Mellitus (DM) [31]. In addition, another meta-analysis of tooth loss and diabetes risk was conducted in recent years with a limited number of studies [32]. Therefore, we conducted the meta-analysis on the number of further studies and subgroup analyses including study type, continent, method of diagnosing diabetes, quality of studies, number of missing teeth, adjusted and unadjusted for confounding factors in calculating effect size in assessing of association between T2D with tooth loss.

## Methods

This systematic review and meta-analysis was reported in according to referred reporting items for systematic reviews and meta-analyses (PRISMA) guideline [33]. This review is not part of a registry for systematic reviews and the protocol has not been published.

### Search strategy and data sources

Literature search were conducted for English evidences in PubMed/Medline, Scopus, ProQuest and Web of Sciences up to February 2022. The following keywords were used: ‘Tooth’ OR ‘teeth’ AND ‘Loss’ AND ‘diabet*’ OR ‘Prediabet*’ OR ‘Glucose Intolerance’. No restriction for publication date was applied. Full search strategies for all sources are listed in Appendix 1. We also performed a manual search of related articles’ references to avoid missing any relevant published papers. Two reviewers independently screened the output of the search to identify potentially eligible studies (M. GH and A.J). Any disagreements between the two reviewers were resolved by the consultation with the principal investigator (A.R).

### Study selection and definition

Each title and abstract were reviewed to identify relevant studies by two individual researchers (M.GH and MM). The full texts of publications were reviewed if the abstract suggested that diabetics had been examined in relation to tooth loss. Studies met the inclusion criteria if: a) had observational design, b) reported odds ratio (OR), prevalence ratio (PR), risk ratio (RR) or hazard ratio (HR) with 95% confidence interval (95% CI) for the category of diabetic or provided number of tooth loss subjects and without tooth loss subjects in each category of diabetic or without diabetic to calculate OR and 95% CI. Studies were excluded if: a) had randomized clinical trial (RCT) design, b) insufficient data studies, animal experiments, letters, case reports and review b) reporting mean (SD), correlation, or regression coefficient as the effect size, and c) were conducted on the same population.

### Data extraction

The following data were extracted from each study: first author’s last name, date of publication, country, study design, duration of follow-up for cohort studies, sex, age, definition of diabetics, type of outcome and its definition, number of total subjects and cases in each category of diabetics, OR, RR, and HR (95% CI) in each category of exposure diabetics and adjusted variables.

### Assessment of the risk of bias and quality of the evidence

Risk of bias of included observational studies were assessed using the Newcastle-Ottawa Scale (NOS) [34, 35]. NOS tool is comprised of three items including: selection, comparability, and outcome. A maximum score of 9 can be awarded to each study. In the current study, the quality of the studies was divided into three categories: low (less than 5 points), moderate (5–6 points) and high (7–8 points).

### Statistical analysis

Stata software version 16 (StataCrop, College Station, Texas, USA) was used to conduct meta-analysis. The ORs (95% CI) were used to calculate summary effect size. Pooled odds ratios were summarized using the Der Simonian and Laird method for random effects models. The reported HRs or RRs and PRs by cohort studies were considered the same as OR. For studies that did not report ORs, HRs, or PRs, we calculated ORs and 95% CIs using relevant formula: OR = (odds of being tooth loss if diabetics / odds of being tooth loss if not diabetics) and 95% CI = exp. [ln (OR) ± 1.96 SE (ln (OR))] [36]. Heterogeneity was assessed based on I2. Subgroup analysis was conducted based on type of study, location, exposure definition, outcome definition, quality of study and adjustment for confounders. Publication bias among included studies was assessed using the visual inspection of funnel plots, Bgge’s and Egger’s regression test. Also, a trim and fill analysis was performed to assess the stability of overall relative risk when the results suggested obvious publication bias.

## Results

### Identification and selections of studies

**Fig. 1:**
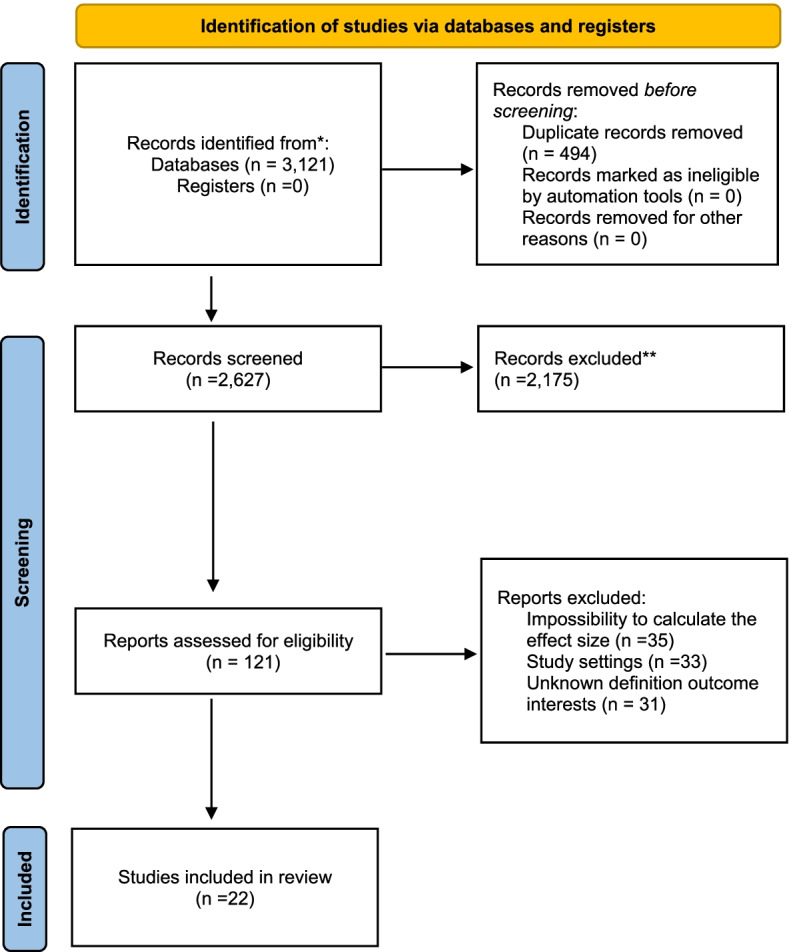
Flow chart of studies reviewed

In the first step of our search, a total of 3121 articles were retrieved using PubMed/Medline, Scopus, Web of Science, ProQuest, and Cochrane library, Google and Google Scholar. Of these articles, 494 articles were removed due to duplication and other 2175 articles were excluded after their title and abstract evaluation. The remaining 121 primary studies were considered for further eligibility assessment through careful reading of their full texts. After full-text evaluation, a further 99 articles were excluded due to inability to calculate effect sizes, study settings, and outcome interest (Table 1). Finally, 22 articles were found to be eligible and included in the systematic review and meta-analysis. The flow chart of study selection process is presented in Fig. 1.

**Table 1:** List of excluded studies

S.NO.	Reference	Reasons for Exclusion
1	Sznajder N, Carraro JJ, Rugna S, Sereday M. Periodontal findings in diabetic and nondiabetic patients. Journal of Periodontology. 1978;49 (9):445–8.	impossibility to calculate the effect size
2	Emrich LJ, Shlossman M, Genco RJ. Periodontal disease in non-insulin-dependent diabetes mellitus. Journal of periodontology. 1991;62 (2):123–31.	impossibility to calculate the effect size
3	Oliver RC, Tervonen T. PERIODONTITIS AND TOOTH LOSS - COMPARING DIABETICS WITH THE GENERAL-POPULATION. Journal of the American Dental Association. 1993;124 (12):71–6.	impossibility to calculate the effect size
4	Hernandez R, Cedola N, Caride E, Pereyra E, Olivera E. Dental pathology in diabetic patients: Absence or loss of teeth. Diabetologia. 1997;40:2356-.	Did not satisfy the outcome criteria
5	Persson RE, Hollender LG, MacEntee MI, Wyatt CCL, Kiyak HA, Persson GR. Assessment of periodontal conditions and systemic disease in older subjects. Focus on diabetes mellitus. Journal of Clinical Periodontology. 2003;30 (3):207–13.	Did not explore the exposure
6	Čuković-Bagić I, Verzak Ž, Car N, Car A. Tooth loss among diabetic patients. Diabetologia Croatica. 2004;33 (1):23–7.	impossibility to calculate the effect size
7	Negishi J, Kawanami M, Terada Y, Matsuhashi C, Ogami E, Iwasaka K, et al. Effect of lifestyle on periodontal disease status in diabetic patients. Journal of the International Academy of Periodontology. 2004;6 (4):120–4.	Did not satisfy the outcome criteria
8	Hu XW, Li CQ, Huang YH, Li XL, Guo F, Li P, et al. Epidemiological survey of the oral diseases of diabetics in west city district in Daqing city. Chinese Journal of Clinical Rehabilitation. 2005;9 (3):28–9.	impossibility to calculate the effect size
9	Mansour AA, Abd-Al-Sada N. Periodontal disease among diabetics in Iraq. MedGenMed: Medscape general medicine. 2005;7 (3):2.	impossibility to calculate the effect size
10	Faggion CM, Petersilka G, Lange DE, Gerss J, Flemmig TF. Prognostic model for tooth survival in patients treated for periodontitis. Journal of Clinical Periodontology. 2007;34 (3):226–31.	Did not explore the exposure
11	Hao JM, Meng HX, Ji LN. The investigation of the periodontal status of type 2 diabetes mellitus families. Zhonghua kou qiang yi xue za zhi = Zhonghua kouqiang yixue zazhi = Chinese journal of stomatology. 2007;42 (7):408–11	Did not satisfy the outcome criteria
12	Demmer R, Phd MPH, Jacobs D, Desvarieux M, Md PHD. Periodontal Disease and Incident Type 2 Diabetes: Results from the First National Health and Nutrition Examination Survey and its Epidemiologic Follow-Up Study. Diabetes Care. 2008;31 (7):1373–9.	Did not satisfy the outcome criteria
13	Leung WK, Shing Chung S, Chu FC, Wong KW, Jin LJ, Sham AS, et al. Oral Health Status of Low-income, Middle-aged to Elderly Hong Kong Chinese with Type 2 Diabetes Mellitus. Oral Health & Preventive Dentistry. 2008;6 (2):105–18.	Did not satisfy the outcome criteria
14	Loe H. The relationship between diabetes and oral health among Australian adults. Australian Dental Journal. 2008;53 (1):93–6.	Did not satisfy the outcome criteria
15	Novak MJ, Potter RM, Blodgett J, Ebersole JL. Periodontal disease in Hispanic Americans with type 2 diabetes. Journal of periodontology. 2008;79 (4):629–36.	Did not satisfy the outcome criteria
16	Patino Marin N, Loyola Rodriguez JP, Medina Solis CE, Pontigo Loyola AP, Reyes Macias JF, Ortega Rosado JC, et al. Caries, periodontal disease and tooth loss in patients with diabetes mellitus types 1 and 2. Acta odontologica latinoamericana: AOL. 2008;21 (2):127–33.	impossibility to calculate the effect size
17	Tanwir F, Altamash M, Gustafsson A. Effect of diabetes on periodontal status of a population with poor oral health. Acta Odontologica Scandinavica. 2009;67 (3):129–33.	Did not satisfy the outcome criteria
18	Silva AM, Vargas AMD, Ferreira E, de Abreu MHNG. Periodontitis in individuals with diabetes treated in the public health system of Belo Horizonte, Brazil. Revista Brasileira de Epidemiologia. 2010;13 (1):118–25.	impossibility to calculate the effect size
19	Stojanovic N, Krunic J, Cicmil S, Vukotic O. Oral Health Status in Patients with Diabetes Mellitus Type 2 in Relation to Metabolic Control of the Disease. Srpski Arhiv Za Celokupno Lekarstvo. 2010;138 (7–8):420–4.	impossibility to calculate the effect size
20	Ueno M, Takeuchi S, Oshiro A, Shinada K, Ohara S, Kawaguchi Y. Association between diabetes mellitus and oral health status in Japanese adults. International journal of oral science. 2010;2 (2):82–9.	Did not satisfy the outcome criteria
21	Kanjirath PP, Kim SE, Rohr Inglehart M. Diabetes and oral health: the importance of oral health-related behavior. Journal of dental hygiene: JDH / American Dental Hygienists’ Association. 2011;85 (4):264–72.	Did not satisfy the study settings
22	Progression of periodontitis in a sample of regular and irregular compliers under maintenance therapy: a 3-year follow-up study	Did not explore the exposure
23	Bajaj S, Prasad S, Gupta A, Singh VB. Oral manifestations in type-2 diabetes and related complications. Indian journal of endocrinology and metabolism. 2012;16 (5):777–9.	Did not satisfy the outcome criteria
24	Botero JE, Yepes FL, Roldán N, Castrillón CA, Hincapie JP, Ochoa SP, et al. Tooth and periodontal clinical attachment loss are associated with hyperglycemia in patients with diabetes. Journal of Periodontology. 2012;83 (10):1245–50	impossibility to calculate the effect size
25	Costa FO, Santuchi CC, Lages EJP, Cota LOM, Cortelli SC, Cortelli JR, et al. Prospective study in periodontal maintenance therapy: comparative analysis between academic and private practices. Journal of periodontology. 2012;83 (3):301–11.	Did not satisfy the study settings
26	Demmer R, Holtfreter B, Desvarieux M, Jacobs D, Kerner W, Nauck M, et al. The Influence of Type 1 and Type 2 Diabetes on Periodontal Disease Progression: Prospective results from the Study of Health in Pomerania (SHIP). Diabetes Care. 2012;35 (10):2036–42.	Did not satisfy the outcome criteria
27	Ochoa SP, Ospina CA, Colorado KJ, Montoya YP, Saldarriaga AF, Miranda Galvis M, et al. [Periodontal condition and tooth loss in diabetic patients]. Biomedica: revista del Instituto Nacional de Salud. 2012;32 (1):52–9.	Did not satisfy the study settings
28	Pei P, Miao L, Zhang M, Zhang ST, Chen YJ. Epidemiological research on factors related to tooth loss of aged population. Chinese Journal of Conservative Dentistry/Yati Yasui Yazhoubingxue Zazhi. 2012;22 (5):292–4.	Did not explore the exposure
29	Batty GD, Li Q, Huxley R, Zoungas S, Taylor BA, Neal B, et al. Oral disease in relation to future risk of dementia and cognitive decline: prospective cohort study based on the Action in Diabetes and Vascular Disease: Preterax and Diamicron Modified-Release Controlled Evaluation (ADVANCE) trial. European psychiatry: the journal of the Association of European Psychiatrists. 2013;28 (1):49–52.	Did not satisfy for both exposure and outcome
30	Huang DL, Chan KCG, Young BA. Poor Oral Health and Quality of Life in Older U.S. Adults with Diabetes Mellitus. Journal of the American Geriatrics Society. 2013;61 (10):1782–8.	Did not satisfy the outcome criteria
31	Kim EK, Lee SG, Choi YH, Won KC, Moon JS, Merchant AT, et al. Association between diabetes-related factors and clinical periodontal parameters in type-2 diabetes mellitus. BMC Oral Health. 2013;13 (1).	Did not satisfy the outcome criteria
32	Al-Khabbaz AK. Type 2 diabetes mellitus and periodontal disease severity. Oral Health and Preventive Dentistry. 2014;12 (1):77–82.	Did not satisfy the outcome criteria
33	Amiri AA, Maboudi A, Bahar A, Farokhfar A, Daneshvar F, Khoshgoeian HR, et al. Relationship between type 2 diabetic retinopathy and periodontal disease in Iranian adults. North American Journal of Medical Sciences. 2014;6 (3):139–44.	Did not satisfy the outcome criteria
34	Azogui-Lévy S, Rochereau T. [Dental health and dental care according diabetic status; results from 2008 ESPS study]. Revue d’epidemiologie et de sante publique. 2014;62 (6):329–37.	Did not satisfy the study settings
35	Cai M, Liang R, Xu Y, Peng H, Wu W. Correlation between tooth loss and chronic disease of elders in Hebei province. Zhengzhou Daxue Xuebao (Yixue Ban) - Journal of Zhengzhou University Medical sciences. 2014;49 (4):577–9.	Did not explore the exposure
36	Passeri CR, Freitas AR, Aznar FD, Caracik J, Peres AS, Peres SCS. IMPACT OF TOOTH LOSS ON QUALITY OF LIFE IN DIABETIC AND NON-DIABETIC MORBIDLY OBESE PATIENTS. Obesity Surgery. 2014;24 (8):1204–5.	Did not explore the exposure
37	Luo H, Pan W, Sloan F, Feinglos M, Wu B. Forty-year trends in tooth loss among american adults with and without diabetes mellitus: An age-period-cohort analysis. Preventing Chronic Disease. 2015;12 (12).	impossibility to calculate the effect size
38	Juncar RI, Juncar M, Popa AR. Oral disease in diabetic patients - A pilot study. Romanian Journal of Diabetes, Nutrition and Metabolic Diseases. 2016;23 (3):247–54.	Did not satisfy the study settings
39	Lo TE, Lagaya-Estrada MC, Jimeno C, Jasul G, Jr. Clinical utility of self-reported oral health measures for predicting periodontitis among adult filipinos with type 2 diabetes mellitus. Journal of the ASEAN Federation of Endocrine Societies. 2016;31 (1):10–7.	Did not satisfy the outcome criteria
40	Padmalatha GV, Bavle RM, Satyakiran GVV, Paremala K, Sudhakara M, Makarla S. Quantification of *Porphyromonas gingivalis* in chronic periodontitis patients associated with diabetes mellitus using real-time polymerase chain reaction. Journal of oral and maxillofacial pathology: JOMFP. 2016;20 (3):413–8.	Did not satisfy the study settings
41	Ramli NIN, Alkaff SNIASMN, Faisal GG, Bayati LHA. Diabetes mellitus; its impact on periodontal health and dental caries. Journal of International Dental and Medical Research. 2016;9 (3):164–8.	Did not satisfy the study settings
42	Schulze A, Busse M. Gender Differences in Periodontal Status and Oral Hygiene of Non-Diabetic and Type 2 Diabetic Patients. Open Dentistry Journal. 2016;10:287–97.	Did not satisfy the outcome criteria
43	Thaper S, Thaper T, Vishnu Priya V, Thaper R, Thaper R. Prevalence of periodontitis in diabetic and non-diabetic patients. Asian Journal of Pharmaceutical and Clinical Research. 2016;9 (1):308–10.	Did not satisfy the outcome criteria
44	Yang BT, Xu JL, He L, Meng HX, Xu L. Porphyromonas gingivalis FimA genotype distribution among periodontitis patients with type 2 diabetes. Zhonghua kou qiang yi xue za zhi = Zhonghua kouqiang yixue zazhi = Chinese journal of stomatology. 2016;51 (1):20–4.	Did not satisfy the study settings
45	Alyasiry AM. Oral Hygiene For The Diabetes Mellitus And Osteoporosis Patients. Research Journal of Pharmaceutical Biological and Chemical Sciences. 2017;8 (3):783–91.	Did not satisfy the outcome criteria
46	Brignardello-Petersen R. Age, sex, diabetes mellitus, and endodontic treatment affect incidence of tooth loss after periodontal treatment. Journal of the American Dental Association. 2017;148 (4):e43.	impossibility to calculate the effect size
47	D’Aiuto F, Gable D, Syed Z, Allen Y, Wanyonyi KL, White S, et al. Evidence summary: The relationship between oral diseases and diabetes. British Dental Journal. 2017;222 (12):944–8.	Did not satisfy the study settings
48	Singh AK, Mishra R. A Prospective Study Establishing Correlation between Diabetes and Tooth Loss. Journal of Advanced Medical and Dental Sciences Research. 2017;5 (12):119A-23A.	impossibility to calculate the effect size
49	Song SJ, Han K, Lee SS, Park JB. Association between the number of natural teeth and diabetic retinopathy among type 2 diabetes mellitus: The Korea national health and nutrition examination survey. Medicine (United States). 2017;96 (47).	Did not explore the exposure
50	Wiener RCMADMDP, Shen CP, Findley PAD, Sambamoorthi UP, Tan XP. The association between diabetes mellitus, sugar-sweetened beverages, and tooth loss in adults: Evidence from 18 states. American Dental Association The Journal of the American Dental Association. 2017;148 (7):500.	impossibility to calculate the effect size
51	Ahmad B, Ahmad O, Afzal M. THE ETIOLOGY OF TOOTH LOSS AND RISK FACTORS CAUSING PERIODONTAL DISEASE. Indo American Journal of Pharmaceutical Sciences. 2018;5 (12):14009–14.	Did not explore the exposure
52	Maia FB, de Sousa ET, Sampaio FC, Freitas CH, Forte FD. Tooth loss in middle-aged adults with diabetes and hypertension: Social determinants, health perceptions, oral impact on daily performance (OIDP) and treatment need. Medicina oral, patologia oral y cirugia bucal. 2018;23 (2):e203-e10.	impossibility to calculate the effect size
53	Oliveira EJP, Roch VFB, Nogueira DA, Pereira AA. Quality of life and oral health among hypertensive and diabetic people in a Brazilian Southeastern city. Ciencia e Saude Coletiva. 2018;23 (3):763–72.	Did not satisfy the outcome criteria
54	Trentin MS, De Carli JP, Ferreira MD, Gambin DJ, da Silva SO, Lisboa H. PREVALENCE AND SEVERITY OF PERIODONTAL DISEASE IN TYPE 2 DIABETES MELLITUS PATIENTS: A CROSS-SECTIONAL STUDY. Bioscience Journal. 2018;34 (4):1114–23.	Did not satisfy the study settings
55	Afolabi O, Adeniyi A, Sofola O, Ogbera A. Effect of glycemic control on periodontal disease and caries experience in diabetic patients: A pilot study. Journal of Interdisciplinary Dentistry. 2019;9 (3):99–107.	Did not satisfy the study settings
56	Glurich I, Acharya A. Updates from the Evidence Base Examining Association between Periodontal Disease and Type 2 Diabetes Mellitus: Current Status and Clinical Relevance. Current Diabetes Reports. 2019;19 (11).	Did not satisfy the study settings
57	Izuora K, Yousif A, Allenback G, Gewelber C, Neubauer M. Relationship between dental loss and health outcomes among hospitalized patients with and without diabetes. Journal of Investigative Medicine. 2019;67 (3):669–73.	impossibility to calculate the effect size
58	Khan SQ, Khabeer A, Al-Thobity AM, Benrashed MA, Alyousef NI, AlMaimouni Y. Correlation between diabetes mellitus and number of restored, carious lesions and missing teeth: A retrospective radiographic evaluation. Saudi Dental Journal. 2020.	impossibility to calculate the effect size
59	Furukawa T, Wakai K, Yamanouchi K, Oshida Y, Miyao M, Watanabe T, et al. Associations of periodontal damage and tooth loss with atherogenic factors among patients with type 2 diabetes mellitus. Intern Med. 2007;46 (17):1359–64.	Did not satisfy the study settings
60	Wu CZ, Yuan YH, Liu HH, Li SS, Zhang BW, Chen W, et al. Epidemiologic relationship between periodontitis and type 2 diabetes mellitus. BMC Oral Health. 2020;20 (1):204.	Did not satisfy the outcome criteria
61	Panezai J, Altamash M, Engstrm PE, Larsson A. Association of glycated proteins with inflammatory proteins and periodontal disease parameters. Journal of Diabetes Research. 2020;2020.	Did not satisfy the outcome criteria
62	Ohtani M, Nishimura T. The preventive and therapeutic application of garlic and other plant ingredients in the treatment of periodontal diseases. Experimental and Therapeutic Medicine. 2020;19 (2):1507–10.	Did not satisfy the study settings
63	Yuan-Jung H, Kun-Der L, Jen-Hao C, Lee M-Y, Ying-Chu L, Feng-Chieh Y, et al. Periodontal Treatment Experience Associated with Oral Health-Related Quality of Life in Patients with Poor Glycemic Control in Type 2 Diabetes: A Case-Control Study. International Journal of Environmental Research and Public Health. 2019;16 (20).	Did not satisfy the study settings
64	Teufer B, Sommer I, Nussbaumer-Streit B, Titscher V, Bruckmann C, Klerings I, et al. Screening for periodontal diseases by non-dental health professionals: a protocol for a systematic review and overview of reviews. Systematic reviews. 2019;8 (1):61.	Did not satisfy the study settings
65	Salmeron D, Garcia FG, Pons-Fuster E, Perez-Sayans M, Lorenzo-Pouso AI, Lopez-Jornet P. Screening for prediabetes and risk of periodontal disease. Diabetes & Metabolic Syndrome-Clinical Research & Reviews. 2019;13 (2):1661–6.	Did not satisfy for both exposure and outcome
66	Pockpa ZAD, Struillou X, Kone D, Mobio GS, Soueidan A, Badran Z. Periodontal Diseases and Age-Related Macular Degeneration: Is There a Link? A Review. The Permanente journal. 2019;23.	Did not satisfy the study settings
67	Kim YT, Choi JK, Kim DH, Jeong SN, Lee JH. Association between health status and tooth loss in Korean adults: Longitudinal results from the National Health Insurance Service-Health Examinee Cohort, 2002–2015. Journal of Periodontal and Implant Science. 2019;49 (3):158–70.	Did not explore the exposure
68	Jin DSS, Liao YT, He L, Meng HX, Li P. [Study on periodontal status of patients with pre-diabetes]. Zhonghua kou qiang yi xue za zhi = Zhonghua kouqiang yixue zazhi = Chinese journal of stomatology. 2019;54 (3):157–63.	Did not satisfy the study settings
69	Andriankaja OM, Joshipura K. Potential association between prediabetic conditions and gingival and/or periodontal inflammation. Journal of Diabetes Investigation. 2014;5 (1):108–14.	Did not satisfy for both exposure and outcome
70	Sima C, Glogauer M. Diabetes mellitus and periodontal diseases. Current Diabetes Reports. 2013;13 (3):445–52.	impossibility to calculate the effect size
71	Leite RS, Marlow NM, Fernandes JK. Oral Health and Type 2 Diabetes. American Journal of the Medical Sciences. 2013;345 (4):271–3.	Did not satisfy the study settings
72	Jiang X, Zhu Y, Liu Z, Tian Z, Zhu S. Association between diabetes and dental implant complications: a systematic review and meta-analysis. Acta Odontologica Scandinavica. 2021;79 (1):9–18.	Did not satisfy the study settings
73	Raju K, Taylor GW, Tahir P, Hyde SS. Association of tooth loss with morbidity and mortality by diabetes status in older adults: a systematic review. Bmc Endocrine Disorders. 2021;21 (1).	Did not satisfy the study settings
74	Carvalho R, Botelho J, Machado V, Mascarenhas P, Alcoforado G, Mendes JJ, et al. Predictors of tooth loss during long-term periodontal maintenance: An updated systematic review. Journal of clinical periodontology. 2021;48 (8):1019–36.	Did not satisfy the study settings
75	Weijdijk LPM, Ziukaite L, Van der Weijden GA, Bakker EWP, Slot DE. The risk of tooth loss in patients with diabetes: A systematic review and meta-analysis. International journal of dental hygiene. 2022;20 (1):145–66.	Did not satisfy the study settings
76	Liljestrand JM, Salminen A, Lahdentausta L, Paju S, Mäntylä P, Buhlin K, et al. Association between dental factors and mortality. 2021;54 (5):672–81.	Did not satisfy for both exposure and outcome
77	Jacob L, Shin JI, Oh H, Lopez-Sanchez GF, Smith L, Haro JM, et al. Association between diabetes and edentulism and their joint effects on health status in 40 low and middle-income countries. Bmj Open Diabetes Research & Care. 2021;9 (1).	Did not satisfy the outcome criteria
78	Altun E, Walther C, Borof K, Petersen E, Lieske B, Kasapoudis D, et al. Association between dietary pattern and periodontitis—a cross-sectional study. Nutrients. 2021;13 (11).	Did not explore the exposure
79	Chatzopoulos GS, Cisneros A, Sanchez M, Wolff LF. Association between Periodontal Disease and Systemic Inflammatory Conditions Using Electronic Health Records: A Pilot Study. Antibiotics (Basel, Switzerland). 2021;10 (4).	Did not satisfy the study settings
80	Menon GR, Malaiappan S, Kumar K. Association between right upper molar involvement and diabetes mellitus in subjects with chronic periodontitis. International Journal of Dentistry and Oral Science. 2021;8 (6):2879–84.	Did not satisfy the outcome criteria
81	Tegelberg P, Tervonen T, Knuuttila M, Jokelainen J, Keinänen-Kiukaanniemi S, Auvinen J, et al. Association of hyperglycaemia with periodontal status: Results of the Northern Finland Birth Cohort 1966 study. Journal of clinical periodontology. 2021;48 (1):24–36.	Did not satisfy the outcome criteria
82	Barma MD, Raj SS, Jayashri P. Clinical Periodontal Parameters Associated with Diabetes Mellitus in Patients Attending a Private Dental Hospital in Chennai. Bioscience Biotechnology Research Communications. 2021;14 (10):83–9.	Did not satisfy the outcome criteria
83	Song TJ, Chang Y, Jeon J, Kim J. Oral health and longitudinal changes in fasting glucose levels: A nationwide cohort study. 2021;16 (6):e0253769.	Did not satisfy the outcome criteria
84	Al-Zahrani MS, Alhassani AA, Zawawi KH. Tooth loss as a potential risk factor for deficient sleep: an analysis of a nationally representative sample of adults in the USA. Journal of public health dentistry. 2021;25 (2):1101–7.	Did not explore the exposure
85	Madi M, Abuohashish HM, Attia D, AlQahtani N, Alrayes N, Pavlic V, et al. Association between Periodontal Disease and Comorbidities in Saudi’s Eastern Province. Biomed Research International. 2021;2021.	Did not satisfy the outcome criteria
86	Yu YH, Cheung WS, Steffensen B, Miller DR. Number of teeth is associated with all-cause and disease-specific mortality. BMC oral health. 2021;21 (1).	Did not explore the exposure
87	Yang LX, Ge Q, Ye ZT, Wang LJ, Wang LP, Mashrah MA, et al. Sulfonylureas for Treatment of Periodontitis-Diabetes Comorbidity-Related Complications: Killing Two Birds With One Stone. Frontiers in Pharmacology. 2021;12.	impossibility to calculate the effect size
88	Tae-Jin S, Chang Y, Jeon J, Kim J. Oral health and longitudinal changes in fasting glucose levels: A nationwide cohort study. PloS one. 2021;16 (6).	impossibility to calculate the effect size
89	Sunakawa Y, Tsugayasu H, Watanabe Y, Matsushita T, Ohara Y, Iwasaki M, et al. Relationship between weight loss and regular dental management of older adults residing in long-term care facilities: a 1-year multicenter longitudinal study. European Geriatric Medicine. 2021.	Did not satisfy for both exposure and outcome
90	Stødle IH, Verket A, Høvik H, Sen A, Koldsland OC. Prevalence of periodontitis based on the 2017 classification in a Norwegian population: The HUNT study. Journal of clinical periodontology. 2021;48 (9):1189–99.	Did not explore the exposure
91	Song TJ, Jeon J, Kim J. Cardiovascular risks of periodontitis and oral hygiene indicators in patients with diabetes mellitus. Diabetes and Metabolism. 2021;47 (6).	Did not satisfy the outcome criteria
92	Shi S, Ding F, Liu X, Wang L, Wang X, Zhang S, et al. Clinical and radiographic variables related to implants with simultaneous grafts among type 2 diabetic patients treated with different hypoglycemic medications: a retrospective study. BMC oral health. 2021;21 (1):214.	Did not satisfy the outcome criteria
93	Shang R, Gao L. Impact of hyperglycemia on the rate of implant failure and peri-implant parameters in patients with type 2 diabetes mellitus: Systematic review and meta-analysis. Journal of the American Dental Association. 2021;152 (3):189–201.e1.	Did not satisfy the study settings
94	Rodakowska E, Jamiolkowski J, Baginska J, Kaminska I, Gabiec K, Stachurska Z, et al. Oral Health–Related Quality of Life and Missing Teeth in an Adult Population: A Cross-Sectional Study from Poland. International journal of environmental research and public health. 2022;19 (3):1626.	Did not explore the exposure
95	Reham Khaled Abou El F, Mona Ahmed Abdel F, Muhammad Ahmed H, Wassel MO, Amira Saad B, Huda Ahmed Amin E, et al. Periodontal diseases and potential risk factors in Egyptian adult population—Results from a national cross-sectional study. PloS one. 2021;16 (11).	Did not satisfy the outcome criteria
96	Raedel M, Noack B, Priess HW, Bohm S, Walter MH. Massive data analyses show negative impact of type 1 and 2 diabetes on the outcome of periodontal treatment. Clinical oral investigations. 2021;25 (4):2037–43.	Did not satisfy the outcome criteria
97	Patel J, Kulkarni S, Doshi D, Poddar P, Srilatha A, Reddy KS. Periodontal disease among non-diabetic coronary heart disease patients. A case-control study. Acta Biomedica. 2021;92 (1):1–11.	Did not satisfy for both exposure and outcome
98	Mukkavilli M, Kulkarni S, Doshi D, Reddy S, Adepu S, Reddy S. Oral health status and self- assessment of oral health risk factors among South Indian diabetic patients: Official Publication of Indian Society for Dental Research. Indian Journal of Dental Research. 2021;32 (2):140–6.	Did not explore the exposure
99	Luo H, Wu B, Kamer AR, Adhikari S, Sloan F, Plassman BL, et al. Oral Health, Diabetes, and Inflammation: Effects of Oral Hygiene Behaviour. International Dental Journal. 2021.	Did not satisfy the outcome criteria

### Characteristics of included articles

The articles included in the meta-analysis comprised 13 cross-sectional study, six cohort studies and three case– control studies. Eight studies were conducted in American countries, seven studies in European and four studies in Asian countries. The publication year of all included articles range from 2007 to 2021. In primary studies, 11 studies the self-report and medical records was used to diagnose of the diabetes and 11 primary studies used glucose level or HbA1c to diagnose of diabetes among participants. In four studies ORs, PRs, RRs (95% CI) were not reported, therefore, we calculated ORs (95% CI) based on relevant formula. In addition, 16 studies have considered the any tooth loss for calculation of the effect sizes, and in six studies tooth loss at least 5 and more considered for calculation of the effect sizes. There were 677,532 participants in the included studies, with the number of participants per study ranging from 60 to 379,021. The characteristics of the included studies are summarized in Table 2. The NOS scores ranged from 3 to 8, with a mean score of 5.81. According to the NOS tool, 17 studies (77.27%) were of moderate or high quality. Tables 3, 4 and 5 provide a detailed summary of the risk of bias for included cohort, case-control and cross-sectional studies using NOS [34, 35].

**Table 2:** Summary of the included studies

Author, publication year	Country	Type of Study	Mean/Median of Age	Sex	Definition of tooth loss	Definition of Diabetes	Sample Size	Adjustment of confounders	OR (95% CI)	aOR (95% CI)
Costa FO, et al., 2013 [37]	Brazil	Case-control	45 (22–71)	Both	any tooth loss	Based on blood sugar	238			5.1 (1.13–9.30)
Deborah L. et al., 2013 [38]	USA	Cross-sectional	74.02 (0.05)	Both	any tooth loss	Self-report	379,021		1.68 (1.55–1.82)	1.25 (1.13–1.37)
Sensorn, W. et al., 2012 [17]	Thailand	Cross-sectional	50.5 (20–86)	Both	any tooth loss	Based on blood sugar	605		1.72 (1.03–2.73)	
Kapp, J. M. et al., 2007	Columbia	Cross-sectional	18–99	Both	any tooth loss	Self-report	155,280		1.64 (1.49–1.80)	1.11 (0.99–1.24)
Yoo, et al. 2019 [15]	South Korea	Cohort	> 18	Both	any tooth loss	Based on blood sugar	10,215	sex, age, and place of residence, social and economic factors	1.29 (1.23–1.36)	1.34 (1.26–1.43)
Dar-Odeh, et al. 2019 [39]	Saudi Arabia.	Cross-sectional	31.2 (10.13)	Women	any tooth loss	Self-report	1768		1.04 (1.02–1.07)	
Frias-Bulhosa, et al. 2018 [28]	Portugal	Case-control	63.8 (12.8)	Both	any tooth loss	Self-report	666		1.65 (0.88–3.14)	
Hastings, et al. 2017 [7]	USA	Cross-sectional	55.8 (3.5)	Male	any tooth loss	Self-report	275	adjusted for same variables as Model 2 plus dental insurance, arthritis, hypertension, and number of medications.	1.43 (0.87–2.38)	
Rai, et al. 2019 [40]	USA	Cross-sectional	55.5 (16.01)	Both	at least tooth loss > = 5	Self-report	1338	CVD, Tobacco use, Age, Gender, Hispanic	1.7 (1.20–2.30)	1.2 (0.80–1.22)
Simila, et al. 2018 [41]	Finland	Cross-sectional	> 46	Women	any tooth loss	Based on blood sugar	5950		3.4 (1.79–6.44)	2.99 (1.54–5.8)
Joshipura, et al. 2018 [42]	USA	Cohort	50.4 (6.8)	Both	at least tooth loss > = 5	Based on blood sugar	1206		1.48 (0.76–2.89)	
Shamala, et al. 2017 [27]	Yemen	Case-control	> 30	Both	at least tooth loss > = 5	Self-report	272		4 (2–8.1)	
Oluwagbemigun, et al. 2015 [26]	Germany	Cohort	51.8 (8.22)	Both	any tooth loss	Self-report	24,313	Age, Sex, BMI, education, occupation, smoking, alcohol consumption, physical activity, use of vitamin and/or mineral supplements, antibiotics, hormone		1.08 (0.81–1.44)
Delgado-Pérez, et al. 2017 [29]	Mexico	Cross-sectional	50.7 (16.2)	Both	any tooth loss		60	replacement therapy (women) and non–steroidal anti–inflammatory drugs	3.42 (2.32–5.04)	3.13 (2.09–4.69)
Buysschaert, et al. 2017 [30]	Belgium	Cross-sectional	62 (15)	Both	at least tooth loss > = 5	Based on blood sugar	160		1.72 (0.85–3.54)	
Liljestrand, et al. 2015 [43]	Finland	Cohort	60.8 (8.44)	Both	at least tooth loss > = 5	Self-report	7629		1.37 (1.02–1.86)	
Kowall, et al. 2015 [44]	Germany	Cross-sectional	57.3 (10.82)	Both	any tooth loss	Based on blood sugar	3086	age, sex, BMI, education, smoking, alcohol consumption, total cholesterol, HDL, cholesterol, and triglycerides	4.02 (3.01–5.36)	1.05 (0.73–1.49)
Greenblatt, et al. 2016 [20]	USA	Cohort	18–74	Both	at least tooth loss > = 5	Based on blood sugar	15,113	age, Hispanic background, study site/center, nativity status, income, and education, number of dental visits and current health insurance status, alternative healthy eating index, cigarette smoking, and obesity, chronic periodontitis	2.69 (2.45–2.97)	1.18 (0.94–1.49)
De Medeiros, et al. 2021 [45]	Brazil	Cross-sectional	> = 30	Both	any tooth loss	Based on blood sugar	60,271	Adults		1.11 (1.08–1.14)
Zhang, et al. 2021 [46]	USA	Cohort	> = 30	Both	any tooth loss	Based on blood sugar	5569	Adults		2.11 (1.46–3.04)
Del Carmen, et al. 2021 [47]	Mexico	Cross-sectional	41.6 (15.4)	Both	any tooth loss	Based on blood sugar	1640	Community Oral Health Program	3.62 (2.86–4.57)	
Laouali, et al. 2021 [48]	France	Cross-sectional	70.17 (6.22)	Women	any tooth loss	Self-report	2857	French national health insurance		1.07 (0.97–1.19)

**Table 3:** Quality assessment using Newcastle-Ottawa Scale for cohort studies^a^

Study	Selection			Comparability		Outcome			Study score
	Representativeness of The exposed cohort	Selection of the non-Exposed cohort	Ascertainment of exposure	Demonstration that the outcome of interest was not present at start of the study	Comparability of Cohorts on the basis of design or analysis	Assessment of outcome	Was follow- up Long enough For the outcome to occur?	Adequacy of follow up of cohorts	
Yoo, et al., 2019	*	*	*	*		*	*	*	7/9
Joshipura, et al. 2018	*	*	*			*	*	*	6/9
Oluwagbemigun, et al. 2015	*	*	*	*	*	*	*		7/9
Liljestrand, et al. 2015	*	*	*			*	*	*	6/9
Greenblatt, et al. 2016	*	*	*	*		*	*	*	7/9
Zhang, et al. 2021	*	*	*	*	*	*	*	*	8/9

^a^Study score less than 4 indicates low quality, a score of 4 to 6 represents poor to moderate, and a score 7 or higher indicates as a good quality

**Table 4:** Quality assessment using Newcastle-Ottawa Scale for case control studies^a^

Study	Selection				Comparability		Exposure			Study score
	case definition adequate	Representativeness of the cases	Selection of Controls	Definition of Controls	study controls for important factors	study controls for other factors	Assessment of exposure	Same method of ascertainment for cases and controls	Non-Response rate	
Costa FO, et al., 2013 [37]	*		*		*	*	*	*		6/9
Frias-Bulhosa, et al. 2018 [28]	*			*			*	*		4/9
Shamala, et al. 2017 [27]	*						*	*		3/9

^a^Study score less than 4 indicates low quality, a score of 4 to 6 represents poor to moderate, and a score 7 or higher indicates as a good quality

**Table 5:** Quality assessment using Newcastle-Ottawa Scale for cross-sectional studies^a^

Study	Selection				Comparability		Outcome		Study score
	Representativeness of the sample	Sample size	Non-respondents	Ascertainment of the exposure	Data/ results adjusted for relevant predictors	Data/results not adjusted for all relevant confounders	Assessment of outcome	Statistical test	
Deborah L. et al., 2013 [38]	*	*					**	*	5/10
Sensorn, W. et al., 2012 [17]				*			**	*	4/10
Kapp, J. M. et al., 2007	*	*	*				**	*	6/10
Dar-Odeh, et al. 2019 [39]	*	*					**	*	5/10
Hastings, et al. 2017 [7]					*	*	**	*	5/10
Rai, et al. 2019 [40]	*	*			*	*	**	*	7/10
Simila, et al. 2018 [41]	*	*		*			**	*	6/10
Delgado-Pérez, et al. 2017 [29]							**	*	3/10
Buysschaert, et al. 2017 [30]				*			**	*	4/10
Kowall, et al. 2015 [44]	*	*		*	*	*	**	*	8/10
De Medeiros, et al. 2021 [45]	*	*		*	*	*	**	*	8/10
Del Carmen, et al. 2021 [47]	*	*		*			**	*	6/10
Laouali, et al. 2021 [48]	*	*			*	*	**	*	7/10

^a^Study score Very Good Studies: 9–10 points, Good Studies: 7–8 points, Satisfactory Studies: 5–6 points, Unsatisfactory Studies: 0 to 4 points

### Meta-analysis

**Fig. 2:**
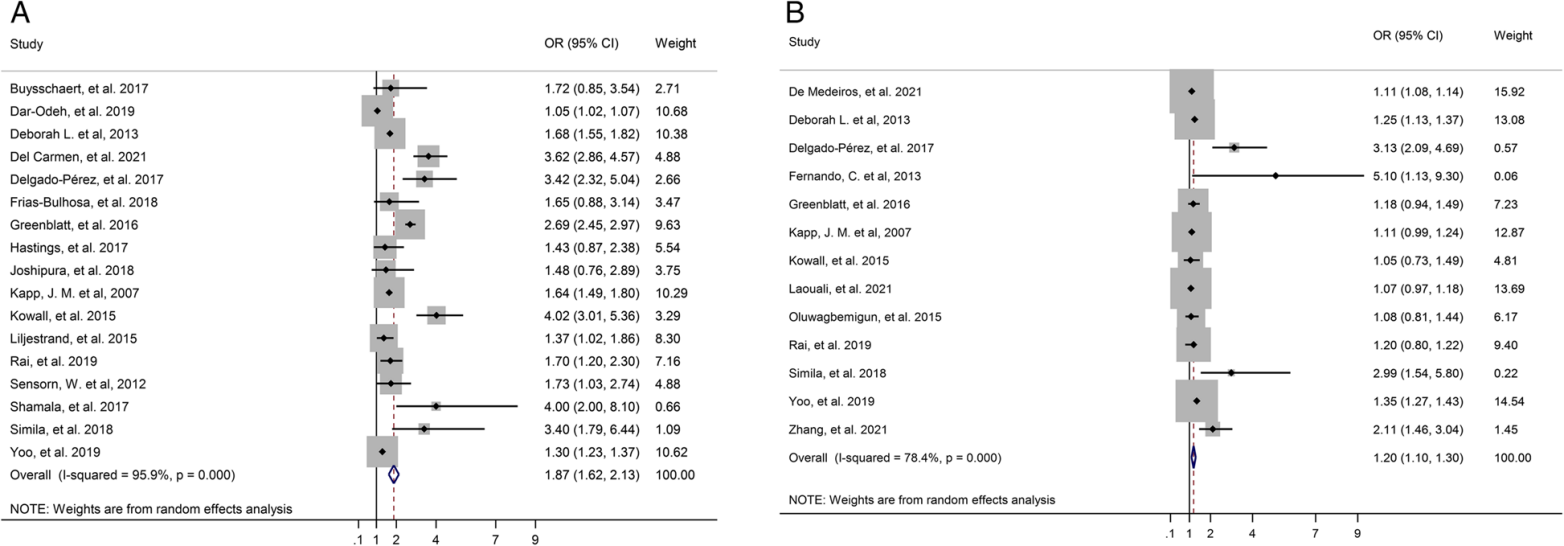
Forest plot of association between T2D and tooth loss. **A**. Unadjusted OR. **B**. Adjusted OR

In the overall summary, in unadjusted and adjusted results indicated that T2D significantly increased the risk of tooth loss, and OR unadjusted was 1.87 (95% CI: 1.62–2.13, *p* < 0.001), and OR adjusted was 1.20 (95% CI: 1.10–1.30, *p* < 0.001), respectively. The forest plot is displayed in Fig. 2.

**Fig. 3:**
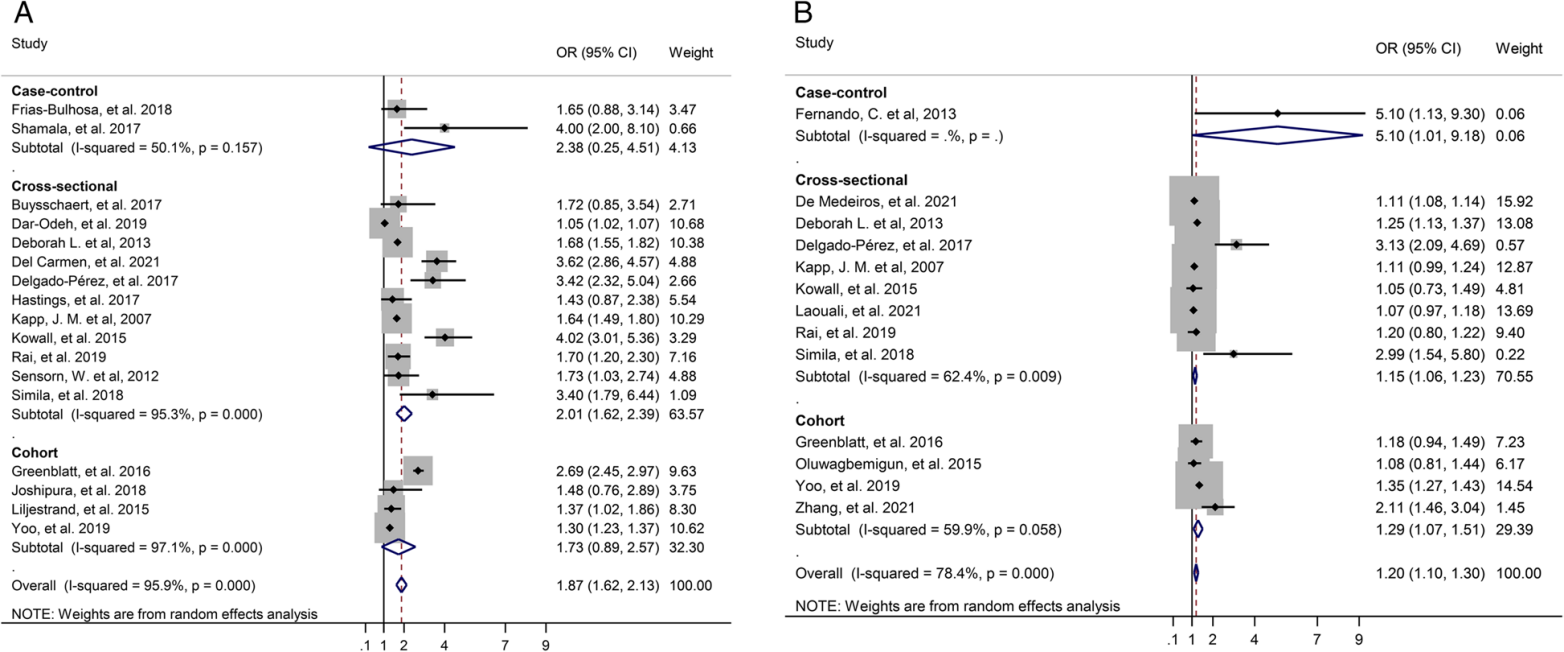
Forest plot of association between T2D and tooth loss by study design. **A**. Unadjusted OR. **B**. Adjusted OR

Subgroup analysis was performed based on the study type, continent, method of diagnosis T2D, category of tooth loss, and quality of study. In the sub-group analysis for unadjusted OR by study type only in the cross-sectional study T2D significantly was associated with tooth loss (OR: 2.01, 95% CI: 1.62–2.39), but in the case-control study (OR: 2.38, 95% CI: 0.25–4.51) and cohort study (OR: 1.73, 95% CI: 0.89–2.57) T2D not significantly was associated with tooth loss. Also, Subgroup analysis based on study design for adjusted OR indicated that in the cohort study (OR: 1.29, 95% CI: 1.07–1.51), in the cross-sectional study (OR: 1.15, 95% CI: 1.06–1.23), and in the case-control study (OR: 5.10, 95% CI: 1.01–9.18) there was a significant association between T2D and tooth loss (Fig. 3).

**Fig. 4:**
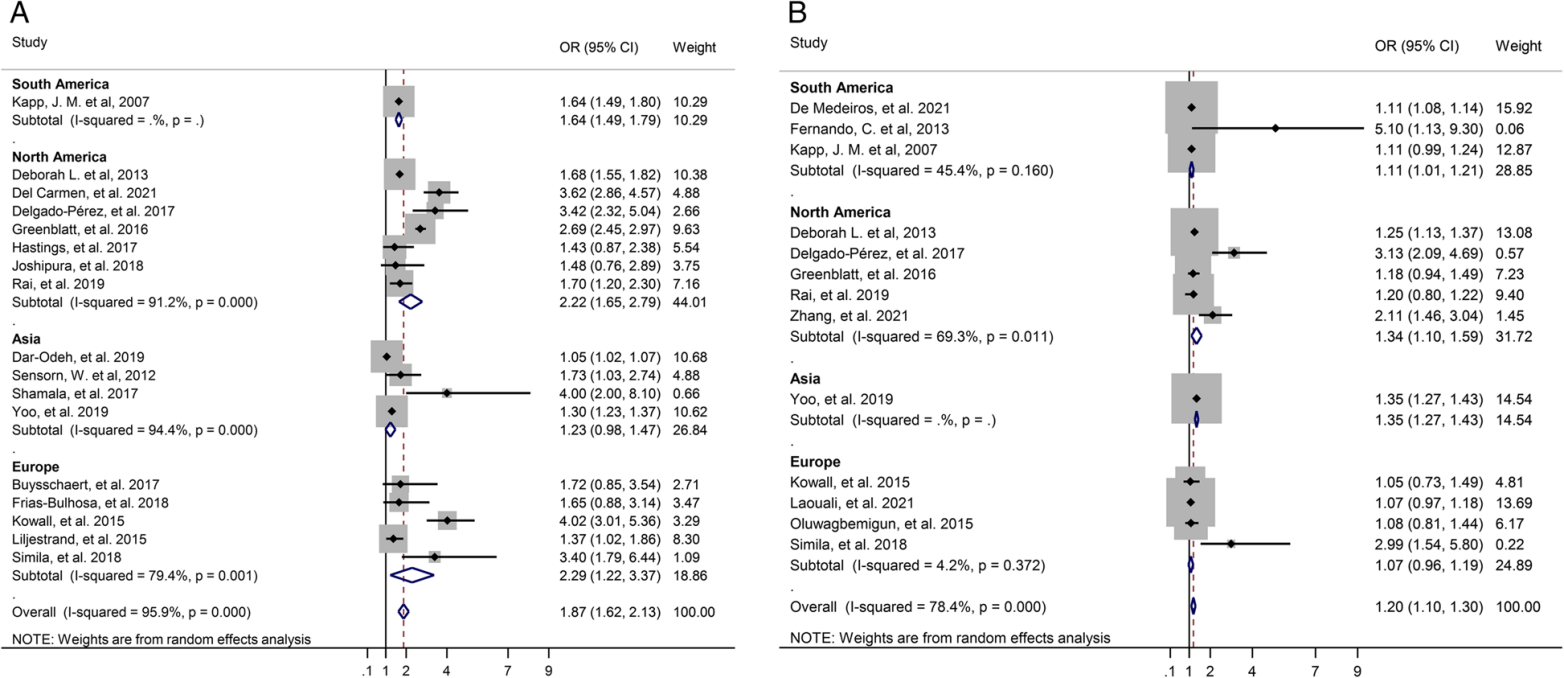
Forest plot of association between T2D and tooth loss by continent. **A**. Unadjusted OR. **B**. Adjusted OR

In addition, in the sub-group analysis for unadjusted OR by continent, were found the T2D associated with tooth loss in North America (OR: 2.22, 95% CI: 1.65–2.79), in South America (OR: 1.64, 95% CI: 1.49–1.79), and in Europe (OR: 2.29, 95% CI: 1.22–3.37), but in Asia, T2D was not significantly associated with tooth loss (OR: 1.23, 95% CI: 0.98–1.47). Also, Subgroup analysis based on the continent for adjusted OR indicated that in North America (OR: 1.34, 95% CI: 1.10–1.59), in South America (OR: 1.11, 95% CI: 1.01–1.21), and in Asia (OR: 1.35, 95% CI: 1.27–1.43) there was a significant association between T2D and tooth loss (Fig. 4).

**Fig. 5:**
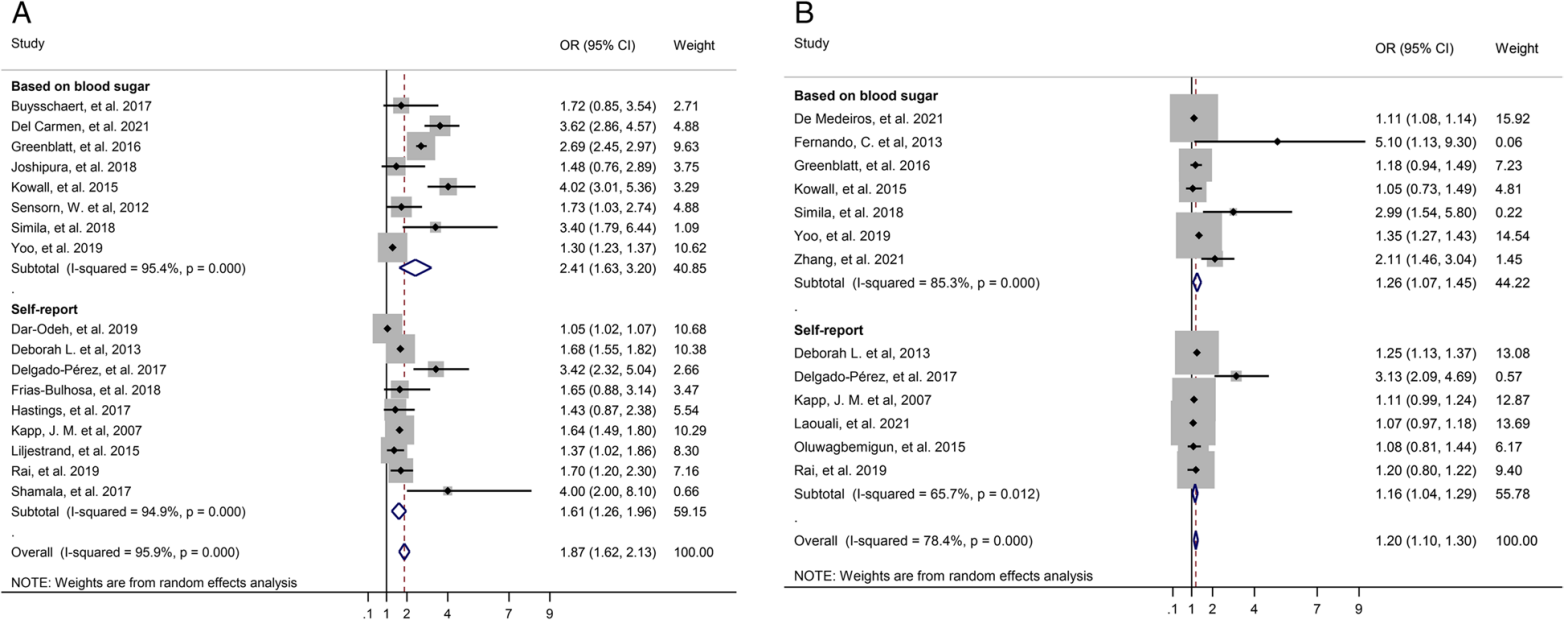
Forest plot of association between T2D and tooth loss by method of diabetes diagnose. **A**. Unadjusted OR. **B**. Adjusted OR

In the subgroup analysis based on the method of diagnosing T2D, we found both in the self-reporting method and in the blood sample detection method a significant association between T2D and tooth loss in unadjusted and adjusted OR. That results showed the association T2D and tooth loss were stronger in the blood sample assessment (Fig. 5).

**Fig. 6:**
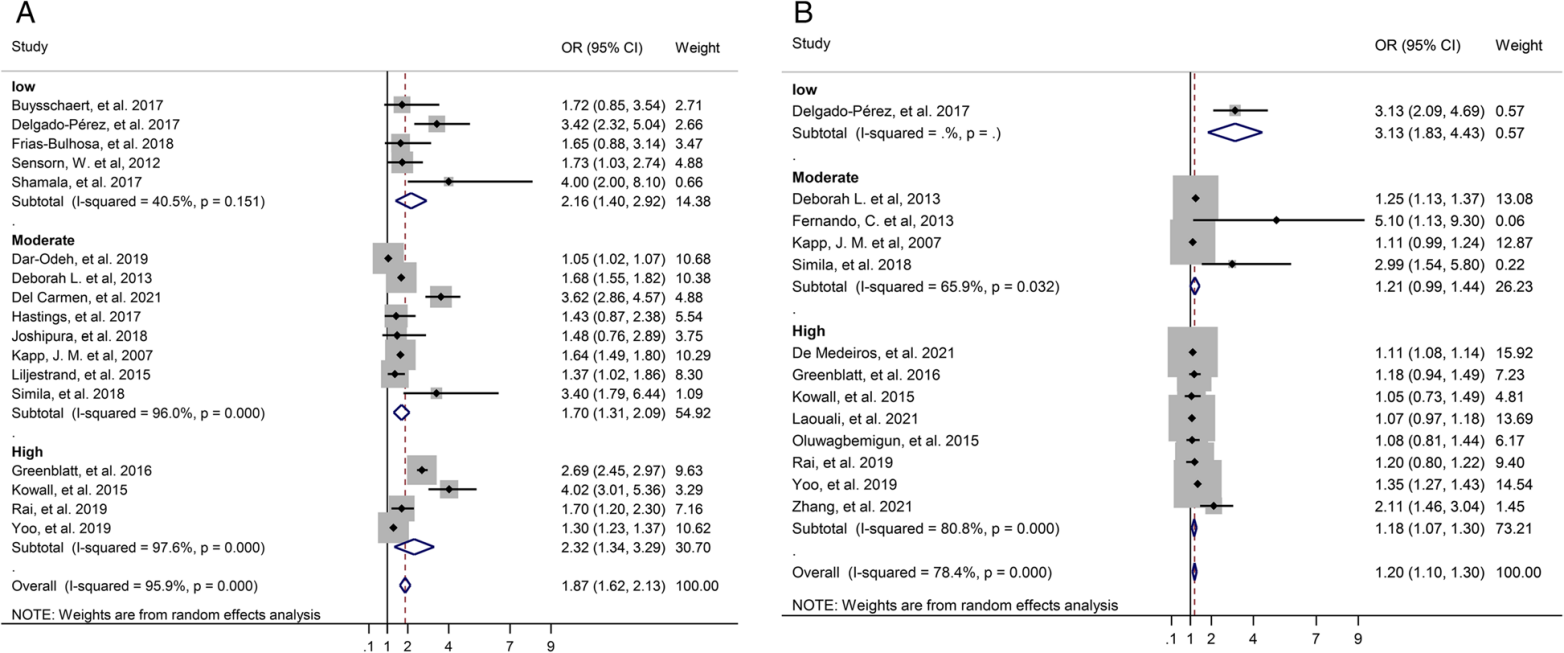
Forest plot of association between T2D and tooth loss by quality of study. **A**. Unadjusted OR. **B**. Adjusted OR

The subgroup analysis for unadjusted OR on quality of studies for those revealed an estimated low quality with an OR of 2.16 and a 95% CI (1.40–2.92), an OR of 1.70 with a 95% CI (1.31–2.09) for those with moderate quality and an OR of 2.32 at a 95% CI (1.34–3.29) for those with high quality. Also, subgroup analysis based on quality of studies for adjusted OR indicated that in the low quality (OR: 3.13, 95% CI: 1.83–4.43), and high quality (OR: 1.18, 95% CI: 1.07–1.30) there was a significant association between T2D and tooth loss, but in moderate quality, T2D was not significantly associated with tooth loss (OR:1.21, 95% CI: 0.99–1.44) (Fig. 6).

**Fig. 7:**
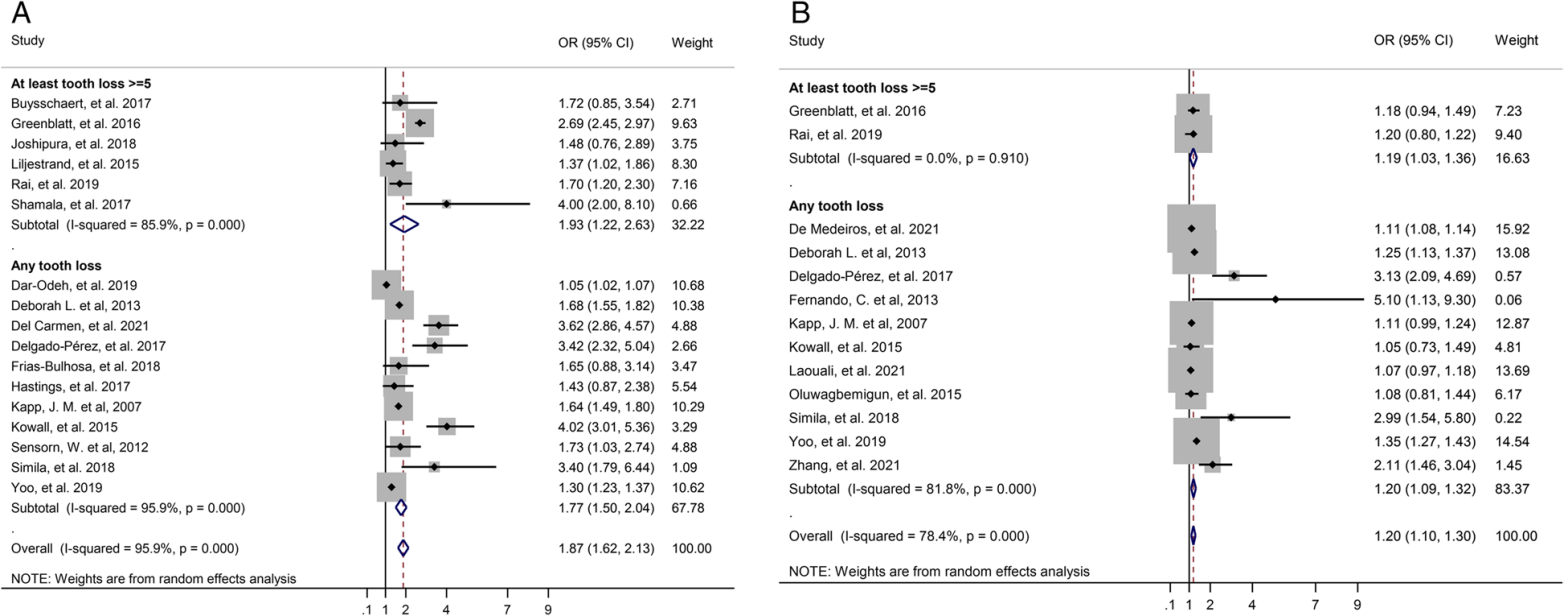
Forest plot of association between T2D and tooth loss by number of tooth loss. **A**. Unadjusted OR. **B**. Adjusted OR

A subgroup analysis for unadjusted OR, on the number of tooth loss was indicated for the category of tooth loss at least 5 teeth and more OR: 1.93 at a 95% CI (1.22–2.63), and any tooth loss OR: 1.77 at a 95% CI (1.50–2.04) Also, subgroup analysis based on the number of tooth loss for adjusted OR indicated that in the category of tooth loss at least 5 teeth and more (OR: 1.19, 95% CI: 1.03–1.36), and for any tooth loss category (OR: 1.20, 95% CI: 1.07–1.32) there was a significant association between T2D and tooth loss (Fig. 7).

### Publication bias

**Fig. 8:**
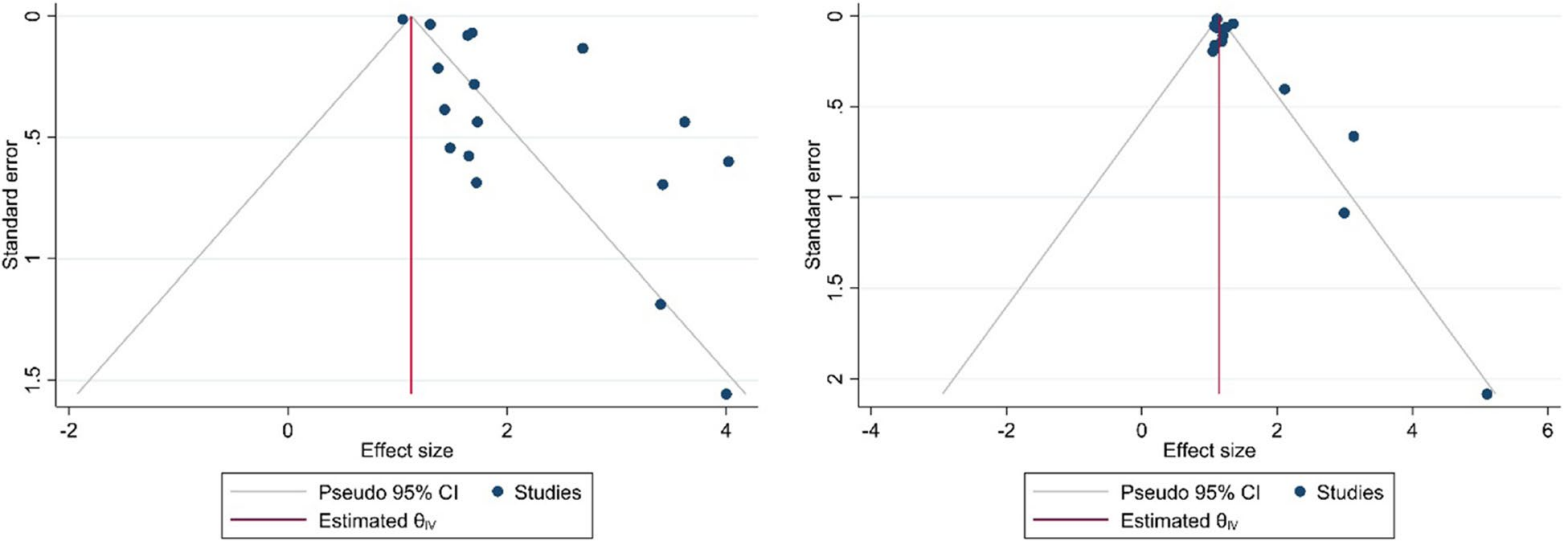
Funnel plot for the association between T2D and tooth loss. **A**. Unadjusted OR. **B**. Adjusted OR

Testing for publication bias was possible for the overall analysis, which is presented in Fig. 8. There seemed to be some publication bias when funnel plots for odds estimates were considered in ORs studies and the *p*-values in the Begg’s test and Egger’s test were *p* = 0.99 and *p* = 0.0001 for unadjusted and the Begg’s test and Egger’s test were *p* = 0.16 and *p* = 0.0005 for adjusted (Fig. 8). However, the trim and fill method for calibration of publication bias was performed because an asymmetry was observed in the visual inspection of the funnel plot. However missing study was not identified by trim and fill method.

## Discussion

The present study summarizes a collection of articles in the field of dentistry and medicine with respect to an important question that examines the relationship between T2D and tooth loss. This systematic review and meta-analysis were conducted on 22 observational studies involving 677,532 participants. The overall summary, indicated that T2D increases the risk of tooth loss by> 1.87 times in unadjusted data and 1.20 times in adjusted data. In addition, this association was also present in other subgroups, including study design, method of diagnosing T2D, continent, study quality, and number of tooth loss. This event seems to be in line with what has been reported in other epidemiologic studies, as several cases have supported the link between diabetes, periodontal disease, and tooth decay [49, 50]. These are two common reasons for the endpoint of the tooth loss parameter. Therefore, the general conclusion for this section is that diabetes maybe can increase the risk of tooth loss.

Review of other meta-analysis studies conducted by Hilal et al. [31] in seven studies that examined the association between diabetes and tooth loss, they found that diabetes was significantly associated with tooth loss (OR: 1.76, 95% CI: 1.21–2.55). Our study with more studies (22 studies) confirmed these findings. In addition, Weijdijk’s et al. [32], which examined the risk of tooth loss in diabetic patients on 10 studies, found that diabetes was significantly associated with the risk of tooth loss (OR: 1.63, 95% CI:1.33–2.00). Our study also confirmed the findings of this study. It should be noted, however, that other studies have not addressed the issue of confounding control in examining the association between diabetes and tooth loss. This is why our adjusted odds ratio (OR:1.20, 95% CI: 1.10–1.30) for association between diabetes and tooth loss is lower than other studies. This indicates an independent association (adjusted for confounders) between diabetes with tooth loss.

Previous observational studies investigating the association between T2D and tooth loss have reported conflicting results. In a cohort study of 15,113 participants, Greenblatt et al. [20] showed that the odds of tooth loss were more than doubled in T2D cases (OR: 2.69, 95% CI: 2.45–2.97). In another cohort study, T2D increased the odds of tooth loss by 29% (OR: 1.29; 95% CI: 1.23–1.36) [15]. However, another cohort study showed no association between T2D and risk of tooth loss [26]. Such findings have also been reported in other types of studies [27–30]. Differences in study design, general characteristics of participants, methodological approach to data collection, diagnosis of T2D, and lack of adjustment for potential confounders may explain the different findings. In a systematic review of 53 observational studies, Wu et al. [51] showed consistent results for the association between T2D and periodontitis. They reported that the prevalence of T2D was significantly higher in patients with periodontitis (OR = 4.04, 95% CI 2.48–6.59, *p* < 0.001) and vice versa (OR = 1.58, 95% CI 1.38–1.81, *p* < 0.001). The findings of this study are consistent with previous evidence that there is a significant association between T2D and tooth loss.

The relationship between diabetes and oral diseases such as tooth loss, gingivitis, periodontal disease, and soft tissue damage has been investigated in various studies [1, 17]. Periodontal disease is the sixth most common complication of diabetes [6, 52]. Patients with T2D have a significantly higher rate of tooth loss. Roughly 22% of diabetics suffer from periodontal disease, which increases with age. Poor blood sugar control can increase the risk of gum problems [10]. There is a two-way relationship between diabetes and periodontitis [17]. Periodontal disease causes high blood sugar, which makes diabetes more difficult to control and makes the patient more susceptible to gum disease [10]. Various studies have shown an association between T2D and periodontal disease [1]. As a result, periodontal disease in diabetics can lead to tooth loss, so diabetics have 15% more tooth loss than non-diabetics [7, 53, 54]. A study showed that the risk of periodontitis in diabetic patients is three times that of non-diabetic patients, and tooth loss is usually caused by periodontal problems. In diabetes, the growth of anaerobic Gram-negative bacteria under the gums is increased [53]. Bacteria in the mouth can form plaques around the teeth and gums. If this plaque is not removed by personal hygiene, the bacteria in the plaque will break down food and produce toxins that cause inflammation of the gums. At this stage, symptoms of gum disease (redness, swelling, bleeding of the gums) appear. If gum disease is not treated, more plaque builds up on the teeth, gums, and under the gums, and this plaque becomes calculus. As a result of the activity of more bacteria, this inflammation can lead to the formation of a gum connection between the tooth and the periodontal pocket. Periodontitis is a stage of the disease that eventually results in the loss of the bone and ligaments surrounding the teeth and loosening as the teeth lose their support, eventually leading to tooth loss [10, 21].

Tooth loss as a proxy for severe periodontitis might play an epidemiologically confusing role in the evaluation of a systemic disease hypothesis [43, 55]. How diabetes leads to periodontal disease and subsequent tooth loss can be explained by various physiological processes, such as immune responses, microflora, cytokines, and glycosylation products. Poorly controlled diabetes can lead to elevated glucose levels in the crevice fluid of the gums in people with diabetes. Therefore, it increases the growth of microaerophilic anaerobic gram-negative bacteria in the areas under the gums [53]. They also impair the function of polymorphonuclear leukocytes (PMNs) and monocytes/macrophages, thereby reducing host defenses against periodontal pathogens and increasing bacterial proliferation. Fat metabolism in diabetics also increases the production of proinflammatory cytokines such as tumor necrosis factor-α (TNF-α) and interleukin-1β (IL-1β) by multinucleated leukocytes and decreases the production of essential polypeptide growth factors such as platelet-derived growth factor (PDGF), transforming growth factor-beta 1 (TGF-β1) and fibroblast growth factor (bFGF) by tissue macrophages [6]. On the other hand, these individuals produce more glycosylation products, which lead to collagen cross-linking, which in addition to reducing collagen solubility, reduces the likelihood of collagen repair and replacement. All of these processes lead to gingivitis, which then progresses to adjacent periodontal tissue, leading to increased tooth loss through increased bone, cement, and ligament damage [15, 54]. Diabetes mellitus (DM) is a chronic inflammatory disease. Evidence supports an increased risk of periodontal disease and the incidence / severity of caries in diabetic patients. Both are major sources of tooth loss.

Between-study heterogeneity is common in meta-analyses, and different characteristics between studies such as year of publication, study location, diagnostic testing, design, and quality of original articles may be sources of between-study heterogeneity [56, 57]. Our meta-analysis revealed significant heterogeneity among studies on T2D and tooth loss risk. Therefore, subgroup analyses did not identify potential factors for heterogeneity between studies.

### Risk of bias

Assessment of risk of bias is a key step in conducting SRs and informs many other steps and decisions within the review. It also plays an important role in the final assessment of the strength of the evidence [58]. Based on the risk of bias of the results, it indicated that all cohort studies included in the selection section met the NOS criteria. However, two of the studies [42, 43] did not adjust for confounders to report results, and results and effect sizes were reported crudely. Among the case-control studies, none of them are representative of cases and the rate of non-response has not been reported. In all cross-sectional studies, tooth loss was assessed using the clinical assessment method, which is the standard method for outcome assessment. Seven [7, 29, 38–40, 48] cross-sectional studies used the self-report method to diagnose diabetes, which increases the risk of bias. Sub-analysis based on the estimated quality of study of the selected studies shows that for low quality, a smaller OR (2.16 and 95% CI [1.40–2.90]) was found than for those with high quality (OR = 2.32 at a 95% CI [1.34–3.29]). Both low-quality and high-quality confidence intervals are small, indicating that the estimates are not flawed by imprecision.

The current systematic reviews and meta-analyses have some strengths and limitations. The study advantages; firstly, our study is the first comprehensive meta-analysis about the association between T2D and tooth loss. Second, in the current study, there was no publication bias between studies, and we performed several subgroup analyses. Third, in the diagnosis of diabetes, the self-report method and the blood sample diagnosis method were evaluated, and the results were reported separately.

There are limitations in our present meta-analysis. First, our study only included articles published in English and the limited number of studies included in this study may lead to false or unstable results. Second, most of the studies included were cross-sectional, so it is difficult to determine the causal relationship between T2D and tooth loss. The results of this study should be interpreted with caution.

## Conclusions

In conclusion, the results of our study showed a positive association between T2D and tooth loss in cross-sectional studies. No significant association between T2D and tooth loss was found in cohort studies. Undoubtedly, large-scale prospective studies are needed to validate the current results in the future.

## Supplementary Information

**Additional file 1.**

## Data Availability

The datasets used and/or analyzed during the current study are available from the corresponding author on reasonable request.

## Abbreviations

T2D: Type 2 Diabetes
CI: Confidence Interval
OR: Odds Ratio
NOS: Newcastle-Ottawa Scale
RR: Relative Ratio
HR: Hazard Ratio
HbA1c: Hemoglobin A1c
PR: Prevalence Ratio
SD: Standard Deviation

## References

[1] Kaur G, Holtfreter B, Rathmann WG, Schwahn C, Wallaschofski H, Schipf S (2009). Association between type 1 and type 2 diabetes with periodontal disease and tooth loss. J Clin Periodontol.

[2] Patiño MN, Loyola R, Medina S, Pontigo L, Reyes M, Ortega R (2007). Caries, periodontal disease and tooth loss in patients with diabetes mellitus types 1 and 2. Acta Odontol Latinoam.

[3] Saeedi P, Petersohn I, Salpea P, Malanda B, Karuranga S, Unwin N (2019). Global and regional diabetes prevalence estimates for 2019 and projections for 2030 and 2045: results from the international diabetes federation diabetes atlas. Diabetes Res Clin Pract.

[4] Williams R, Karuranga S, Malanda B, Saeedi P, Basit A, Besançon S (2020). Global and regional estimates and projections of diabetes-related health expenditure: results from the international diabetes federation diabetes atlas. Diabetes Res Clin Pract.

[5] Kapp JM, Boren SA, Yun S, LeMaster J. Peer reviewed: diabetes and tooth loss in a national sample of dentate adults reporting annual dental visits. Prev Chronic Dis. 2007;4(3):A59. PMC1955413 17572963

[6] Furukawa T, Wakai K, Yamanouchi K, Oshida Y, Miyao M, Watanabe T (2007). Associations of periodontal damage and tooth loss with atherogenic factors among patients with type 2 diabetes mellitus. Intern Med.

[7] Hastings JF, Vasquez E (2017). Diabetes and tooth loss among working-age African Americans: a national perspective. Social Work in Public Health.

[8] Vidone L (2018). A healthy mouth: an important part of a diabetes management plan. AADE Pract.

[9] Wilder RS, Moretti AJ (2021). Overview of gingivitis and periodontitis in adults.

[10] Manish K, Raman N, Gautam A, Jain S, Jha PC, Kumar A. A study on association 0f tooth loss & periodontal disease in patients suffering from diabetes from bihar region. Int J Med Biomed Stud. 2020;4(2):220–6.

[11] Al Habashneh R, Khader Y, Hammad MM, Almuradi M (2010). Knowledge and awareness about diabetes and periodontal health among Jordanians. J Diabetes Complicat.

[12] Control CfD, Prevention (2005). Dental visits among dentate adults with diabetes--United States, 1999 and 2004. MMWR Morb Mortal Wkly Rep.

[13] Jansson H, Lindholm E, Lindh C, Groop L, Bratthall G (2006). Type 2 diabetes and risk for periodontal disease: a role for dental health awareness. J Clin Periodontol.

[14] Ogunbodede E, Fatusi O, Akintomide A, Kolawole K, Ajayi A (2005). Oral health status in a population of Nigerian diabetics. J Contemp Dent Pract.

[15] Yoo JJ, Kim DW, Kim MY, Kim YT, Yoon JH (2019). The effect of diabetes on tooth loss caused by periodontal disease: a nationwide population-based cohort study in South Korea. J Periodontol.

[16] Thorstensson H, Johansson B (2010). Why do some people lose teeth across their lifespan whereas others retain a functional dentition into very old age?. Gerodontology.

[17] Sensorn W, Chatrchaiwiwatana S, Bumrerraj S (2012). Relationship between diabetes mellitus and tooth loss in adults residing in Ubonratchathani province, Thailand. J Med Assoc Thai.

[18] Patel MH, Kumar JV, Moss ME (2013). Diabetes and tooth loss: an analysis of data from the National Health and nutrition examination survey, 2003–2004. J Am Dent Assoc.

[19] Wiener RC, Shen C, Findley PA, Sambamoorthi U, Tan X (2017). The association between diabetes mellitus, sugar-sweetened beverages, and tooth loss in adults: evidence from 18 states. J Am Dent Assoc.

[20] Greenblatt AP, Salazar CR, Northridge ME, Kaplan RC, Taylor GW, Finlayson TL (2016). Association of diabetes with tooth loss in Hispanic/Latino adults: findings from the Hispanic Community Health Study/Study of Latinos. BMJ Open Diabetes Res Care.

[21] Broadbent J, Thomson W, Poulton R (2006). Progression of dental caries and tooth loss between the third and fourth decades of life: a birth cohort study. Caries Res.

[22] Deguchi M, Mau MKLM, Davis J, Niederman R. Peer reviewed: preventable tooth loss in Hawai ‘i: the role of socioeconomic status, diabetes, and dental visits. Prev Chronic Dis. 2017;14:E115. 10.5888/pcd14.170214 PMC5695642 29144892

[23] Felton DA (2009). Edentulism and comorbid factors. J Prosthodont.

[24] Harada K, Morino K, Ishikawa M, Miyazawa I, Yasuda T, Hayashi M (2020). Impact of glycaemic control on the number of teeth remaining: a cross-sectional analysis using a database containing japanese employment-based health insurance and check-up data.

[25] Joshipura KJ, Ritchie C (2005). Can the relation between tooth loss and chronic disease be explained by socio-economic status?. Eur J Epidemiol.

[26] Oluwagbemigun K, Dietrich T, Pischon N, Bergmann M, Boeing H. Association between number of teeth and chronic systemic diseases: a cohort study followed for 13 years. Plos One. 2015;10(5):e0123879. 10.1371/journal.pone.0123879 PMC4422697 25945503

[27] Shamala A, Al-Hajri M, Al-Wesabi MA (2017). Risk factors for periodontal diseases among yemeni type II diabetic patients. A case-control study. J Oral Res.

[28] Frias-Bulhosa J, Manso MC, Mota CL, Melo P (2018). Self-rated health and oral health in type 2 diabetic patients - a case-control study. Revista Portuguesa De Estomatologia Medicina Dentaria E Cirurgia Maxilofacial.

[29] Delgado-Pérez VJ, De La Rosa-Santillana R, Márquez-Corona ML, Ávila-Burgos L, Islas-Granillo H, Minaya-Sánchez M (2017). Diabetes or hypertension as risk indicators for missing teeth experience: an exploratory study in a sample of Mexican adults. Niger J Clin Pract.

[30] Buysschaert M, Muhindo CT, Alexopoulou O, Rahelic D, Reychler H, Preumont V (2017). Oral hygiene behaviours and tooth-loss assessment in patients with diabetes: a report from a diabetology Centre in Belgium. Diabetes Metab.

[31] Helal O, Goestemeyer G, Krois J, Fawzy El Sayed K, Graetz C, Schwendicke F (2019). Predictors for tooth loss in periodontitis patients: systematic review and meta-analysis. J Clin Periodontol.

[32] Weijdijk LP, Ziukaite L, Van der Weijden G, Bakker EW, Slot DE. The risk of tooth loss in patients with diabetes: a systematic review and meta-analysis. Int J Dent Hyg. 2022;20(1):145–66. 10.1111/idh.12512 PMC9291053 33973353

[33] Page MJ, McKenzie JE, Bossuyt PM, Boutron I, Hoffmann TC, Mulrow CD (2021). Updating guidance for reporting systematic reviews: development of the PRISMA 2020 statement. J Clin Epidemiol.

[34] Peterson J, Welch V, Losos M, Tugwell P (2011). The Newcastle-Ottawa scale (NOS) for assessing the quality of nonrandomised studies in meta-analyses.

[35] Modesti PA, Reboldi G, Cappuccio FP, Agyemang C, Remuzzi G, Rapi S (2016). Panethnic differences in blood pressure in Europe: a systematic review and Meta-analysis. Plos One.

[36] Hamling J, Lee P, Weitkunat R, Ambühl M (2008). Facilitating meta-analyses by deriving relative effect and precision estimates for alternative comparisons from a set of estimates presented by exposure level or disease category. Stat Med.

[37] Costa FO, Santuchi CC, Lages EJP, Cota LOM, Cortelli SC, Cortelli JR (2012). Prospective study in periodontal maintenance therapy: comparative analysis between academic and private practices. J Periodontol.

[38] Huang DL, Chan KCG, Young BA (2013). Poor oral health and quality of life in older U.S. adults with diabetes mellitus. J Am Geriatr Soc.

[39] Dar-Odeh N, Borzangy S, Babkair H, Farghal L, Shahin G, Fadhlalmawla S, et al. Association of dental caries, retained roots, and missing teeth with physical status, diabetes mellitus and hypertension in women of the reproductive age. Int J Environ Res Public Health. 2019;16(14):2565. 10.3390/ijerph16142565 PMC6678296 31323793

[40] Rai NK, Carey C, Brunson D, Tiwari T (2019). Increasing dental Students’understanding of population surveillance through data mining. J Dent Educ.

[41] Simila T, Auvinen J, Puukka K, Keinanen-Kiukaanniemi S, Virtanen JI (2018). Impaired glucose metabolism is associated with tooth loss in middle-aged adults: the Northern Finland birth cohort study 1966. Diabetes Res Clin Pract.

[42] Joshipura KJ, Munoz-Torres FJ, Dye BA, Leroux BG, Ramirez-Vick M, Perez CM (2018). Longitudinal association between periodontitis and development of diabetes. Diabetes Res Clin Pract.

[43] Liljestrand JM, Havulinna AS, Paju S, Männistö S, Salomaa V, Pussinen PJ (2015). Missing teeth predict incident cardiovascular events, diabetes, and death. J Dent Res.

[44] Kowall B, Holtfreter B, Volzke H, Schipf S, Mundt T, Rathmann W (2015). Pre-diabetes and well-controlled diabetes are not associated with periodontal disease: the SHIP trend study. J Clin Periodontol.

[45] de Medeiros TCC, Areas E, Souza A, Prates RC, Chapple I, Steffens JP. Association between tooth loss, chronic conditions, and common risk factors—results from the 2019 Brazilian health survey. J Periodontol. 2021; Online ahead of print. 10.1002/JPER.21-0433 34904717

[46] Zhang S, Philips KH, Moss K, Wu D, Adam HS, Selvin E (2021). Periodontitis and risk of diabetes in the atherosclerosis risk in communities (ARIC) study: A BMI-modified association. J Clin Endocrinol Metab.

[47] Fatima del Carmen AD, Aída BYS, Javier DLFH (2021). Risk indicators of tooth loss among Mexican adult population: A cross-sectional study. Int Dent J.

[48] Laouali N, El Fatouhi D, Aguayo G, Balkau B, Boutron-Ruault MC, Bonnet F, et al. Type 2 diabetes and its characteristics are associated with poor oral health: findings from 60,590 senior women from the E3N study. BMC Oral Health. 2021;21(1):315. 10.1186/s12903-021-01679-w PMC8220760 34162373

[49] Chapple IL, Genco R, workshop* wgotjEA (2013). Diabetes and periodontal diseases: consensus report of the joint EFP/AAP workshop on periodontitis and systemic diseases. J Periodontol.

[50] Chapple IL, Bouchard P, Cagetti MG, Campus G, Carra MC, Cocco F (2017). Interaction of lifestyle, behaviour or systemic diseases with dental caries and periodontal diseases: consensus report of group 2 of the joint EFP/ORCA workshop on the boundaries between caries and periodontal diseases. J Clin Periodontol.

[51] Wu CZ, Yuan YH, Liu HH, Li SS, Zhang BW, Chen W (2020). Epidemiologic relationship between periodontitis and type 2 diabetes mellitus. BMC Oral Health.

[52] Malviya M, Trivedi M, Chourasia PK, Tote JV. A study of association between diabetes mellitus and tooth loss among diabetic patients. Glob J Res Analysis (GJRA). 2019;8(11):2277–8160.

[53] Singh AK, Mishra R (2017). A prospective study establishing correlation between diabetes and tooth loss. J Adv Med Dent Sci Res.

[54] Preshaw P, Alba A, Herrera D, Jepsen S, Konstantinidis A, Makrilakis K (2012). Periodontitis and diabetes: a two-way relationship. Diabetologia.

[55] Desvarieux M, Demmer RT, Rundek T, Boden-Albala B, Jacobs DR, Papapanou PN (2003). Relationship between periodontal disease, tooth loss, and carotid artery plaque: the Oral infections and vascular disease epidemiology study (INVEST). Stroke.

[56] Higgins JP, Thompson SG, Deeks JJ, Altman DG (2003). Measuring inconsistency in meta-analyses. BMJ.

[57] Higgins J, Thompson S, Deeks J, Altman D (2002). Statistical heterogeneity in systematic reviews of clinical trials: a critical appraisal of guidelines and practice. J Health Serv Res Policy.

[58] Viswanathan M, Ansari M, Berkman N, Chang S, Hartling L, McPheeters M, et al. Assessing the risk of bias of individual studies in systematic reviews of health care interventions. Agency for healthcare research and quality methods guide for comparative effectiveness reviews. US: AHRQ Methods for Effective Health Care; 2012. 22479713

