# Supplementary material for: Association between type 2 diabetes (T2D) and tooth loss: a systematic review and meta-analysis
Source: BMC Endocr Disord. 2022 Apr 13;22:100. doi: 10.1186/s12902-022-01012-8 (PMC9006550; doi:10.1186/s12902-022-01012-8)
Supplement: Supplementary file 1 — Additional file 1. [file 12902_2022_1012_MOESM1_ESM.docx]

**February 2022**

**Main Syntax:**

**((****Tooth OR teeth) AND Loss) AND (diabet* OR Prediabet* OR Glucose Intolerance**)

| **Database** | **Formula** | **No. of Results** |
| --- | --- | --- |
| **PubMed** | ((((Tooth Loss[Title/Abstract] OR teeth Loss[Title/Abstract])) AND (diabet*[Title/Abstract] OR Prediabet*[Title/Abstract] OR Glucose Intolerance[Title/Abstract]))) OR (("Diabetes Mellitus"[Mesh]) AND "Tooth Loss"[Mesh]) | 480 |
| **Scopus** | ( ( TITLE-ABS-KEY ( prediabetic ) OR TITLE-ABS-KEY ( diabet* ) OR TITLE-ABS-KEY ( glucose AND intolerance ) ) ) AND ( ( TITLE-ABS-KEY ( teeth AND loss ) ) OR ( TITLE-ABS-KEY ( tooth AND loss ) ) ) | 1,507 |
| **Web of Science** | TOPIC: (Tooth Loss OR teeth Loss) AND TOPIC: (diabet* OR Prediabet* OR Glucose Intolerance)  Timespan: All years. Indexes: SCI-EXPANDED, SSCI, A&HCI, CPCI-S, CPCI-SSH, BKCI-S, BKCI-SSH, ESCI, CCR-EXPANDED, IC. | 895 |
| **ProQuest** | ab(((Tooth OR teeth) AND Loss) AND (diabet* OR Prediabet* OR Glucose Intolerance)) OR su(((Tooth OR teeth) AND Loss) AND (diabet* OR Prediabet* OR Glucose Intolerance)) | 237 |
| **Cochrane** | (Tooth Loss OR teeth Loss in Title Abstract Keyword AND diabet* OR Prediabet* OR Glucose Intolerance in Title Abstract Keyword - (Word variations have been searched)) OR (MeSH descriptor: [Tooth Loss] explode all trees AND [Diabetes Mellitus] explode all trees) | 2 |
